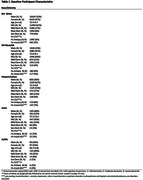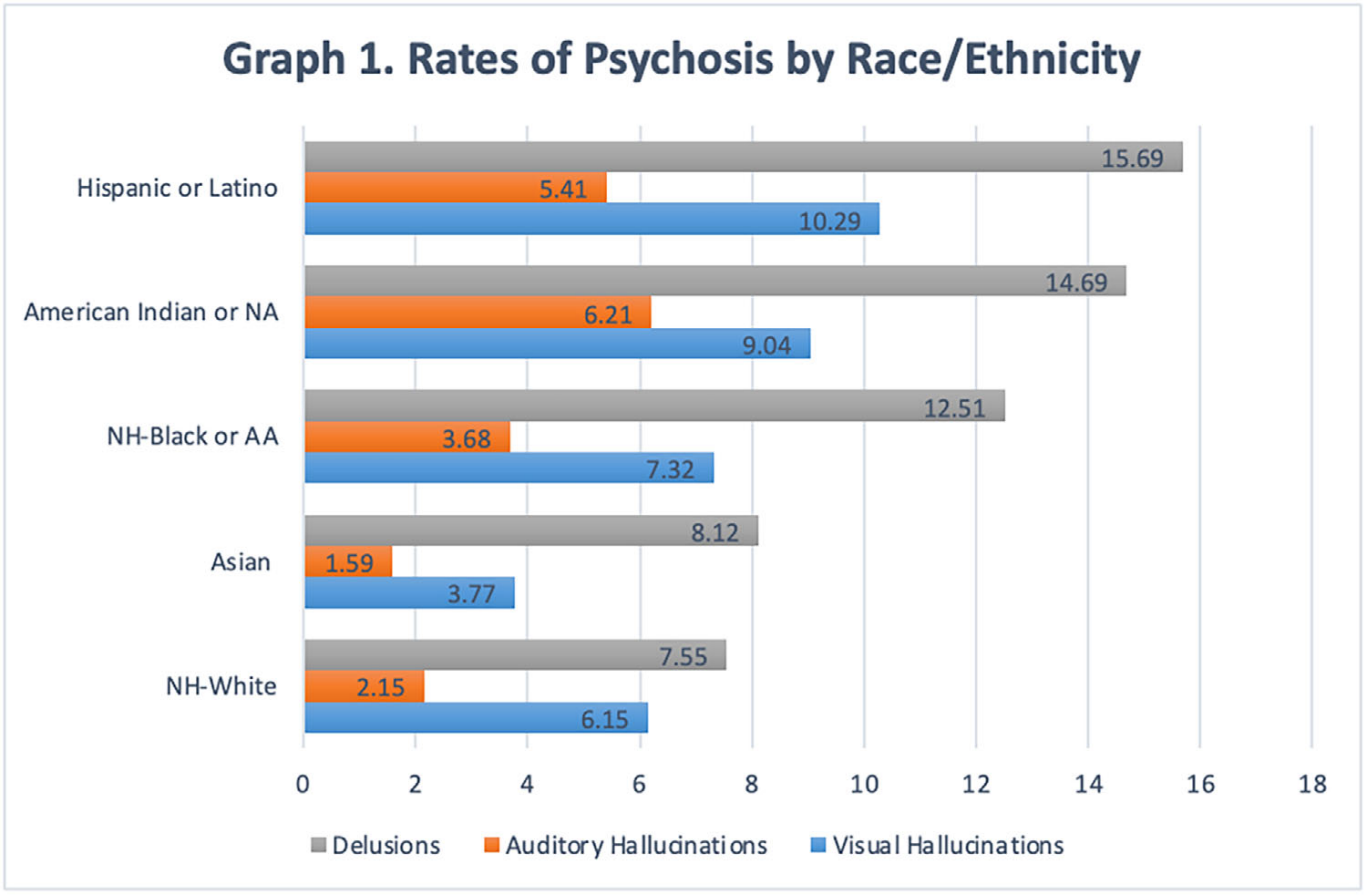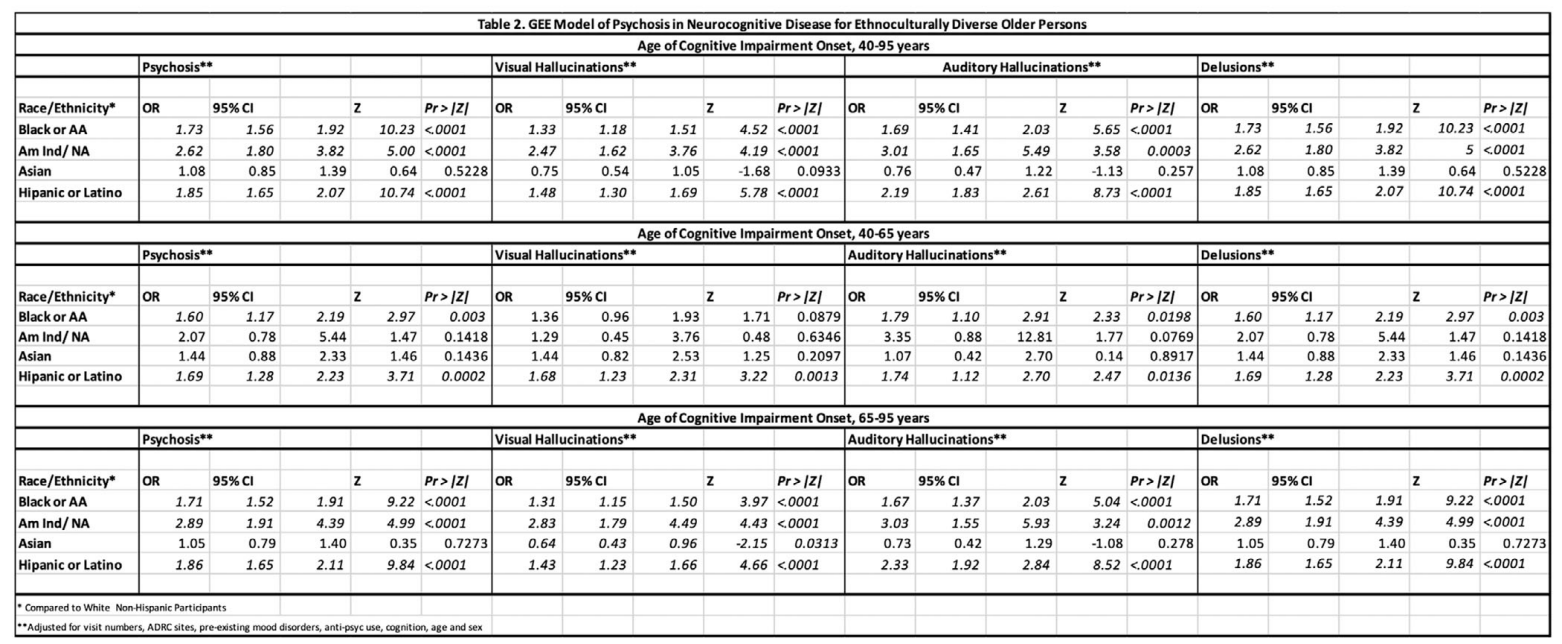# Psychosis in Neurocognitive Disorder for Ethnoculturally Diverse Older Persons

**DOI:** 10.1002/alz.087144

**Published:** 2025-01-03

**Authors:** Estevana Juanita Isaac, Alex Federman, Mary Sano, Albert Siu, Monica Rivera Mindt, Carolyn W. Zhu

**Affiliations:** ^1^ Icahn School of Medicine, Mount Sinai Hospital, New York, NY USA

## Abstract

**Background:**

There is longstanding evidence that the presence of psychosis in neurocognitive disorders is associated with faster deterioration of cognitive function. These reports also describe greater care partner burden, higher rates of institutionalization and functional decline, especially among ethnoculturally diverse persons. **The goal of this study is to examine the association of race/ethnicity with rates of psychosis in neurocognitive disorders among ethnoculturally diverse older persons**.

**Method:**

Data are from the National Alzheimer’s Coordinating Center Uniform Dataset, a longitudinal dataset with annual patient assessments from ∼40 National Institute of Aging funded Alzheimer’s Disease Research Centers. Participants aged 40‐95 years as of June 2023 with mild cognitive impairment (MCI) or dementia were included. Psychosis was defined as clinician diagnosed visual or auditory hallucinations or delusions. Race and ethnicity were self‐reports categorized as non‐Hispanic White (NHW), NH‐Black/African American (AA), Hispanic/Latino, Asian, and American Indian/Native Alaskan (AI/NA). Associations between race/ethnicity and psychosis were estimated using multivariable generalized estimating equation (GEE) models with repeated measures of outcomes and covariates (severity of cognitive impairment, age, sex, history of anti‐psychotic medication use and history of mood disorder). Analyses stratified by age of cognitive impairment onset (40‐65 years and 65‐95 years) were also performed.

**Result:**

25,731 participants had a mean age 72.5±9.7, 51% female, 76% NHW, 12% NH‐Black/AA, 8% Hispanic/Latino, 3% Asian and 1% AI/NA (Table 1). At baseline, 15,646 (60.8%) had MCI and 10,085 (39.2%) had dementia. The rate of psychosis was highest among Hispanic/Latino (15.7%), AI/NA (14.7%) and Black/AA (12.5%) (Graph1). In adjusted analysis, NH‐Black/AA had significantly greater odds of psychosis (OR 1.73, 95% CI 1.17‐2.19), including visual hallucinations (OR 1.33, 95% CI 1.18‐1.51), auditory hallucinations (OR 1.69, 95% CI 1.41‐2.03) and delusions (OR 1.73, 95% CI 1.56‐1.92) (*p<0.05*) (Table 2). Hispanic/Latino and AI/NA participants had even greater odds of psychosis than NHWs (Table 2). Only Black/AA and Hispanic/Latino participants with early‐onset neurocognitive disorder (40‐65 years) had greater odds of psychosis than NHWs (Table 2).

**Conclusion:**

Black/AA, Hispanic/Latino and AI/NA individuals were more likely to be diagnosed with psychosis in neurocognitive disorders when compared to NHWs. More research is needed to explore psychosocial, neuropathology and genetic factors involved.